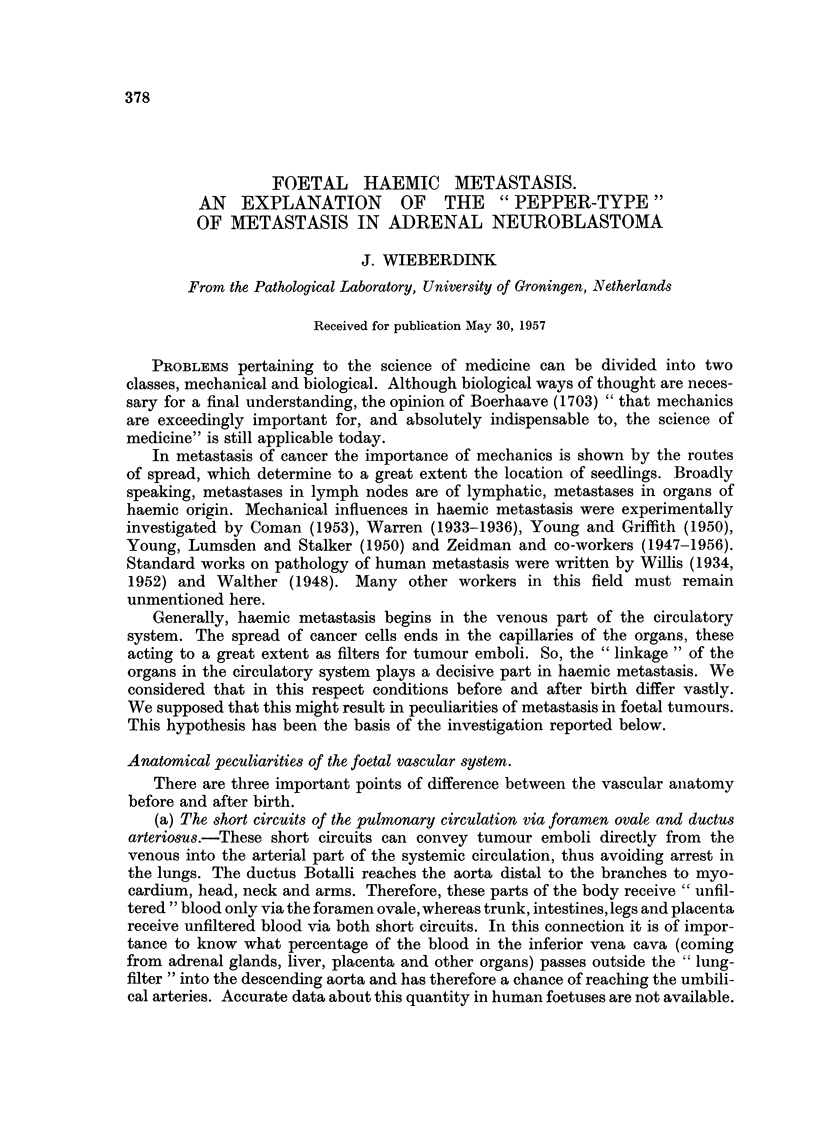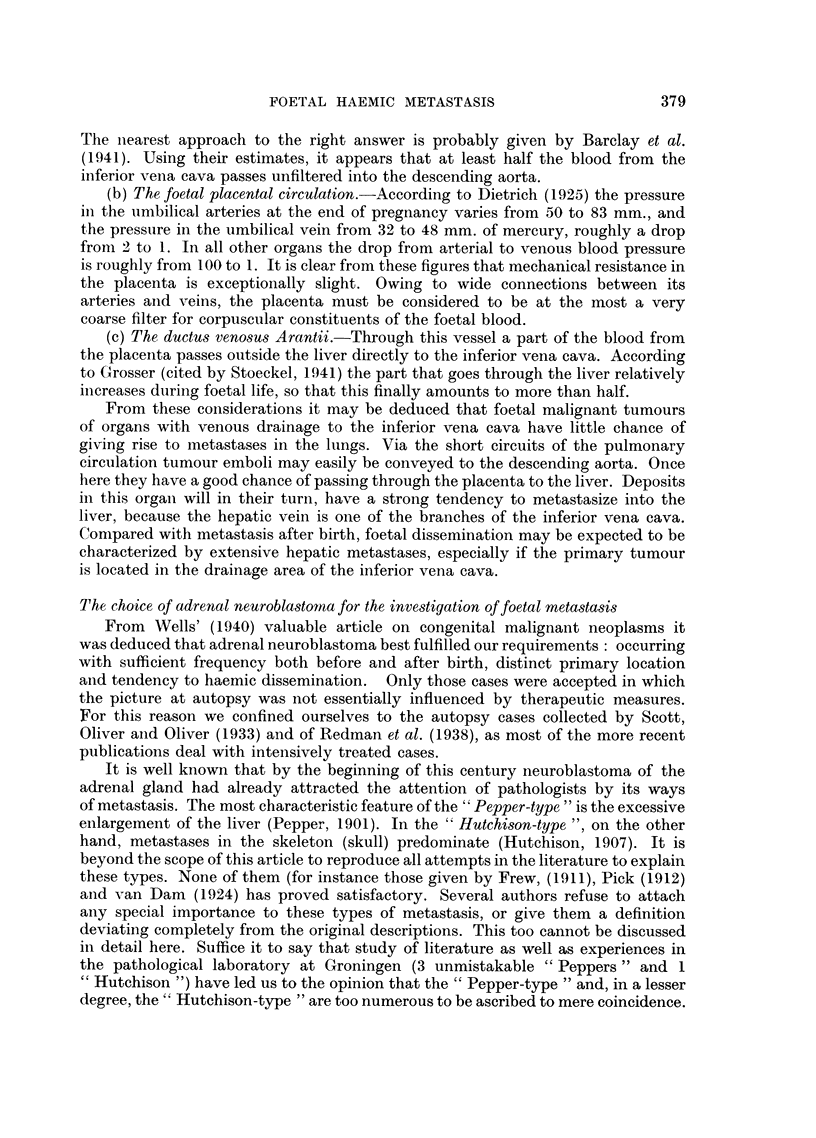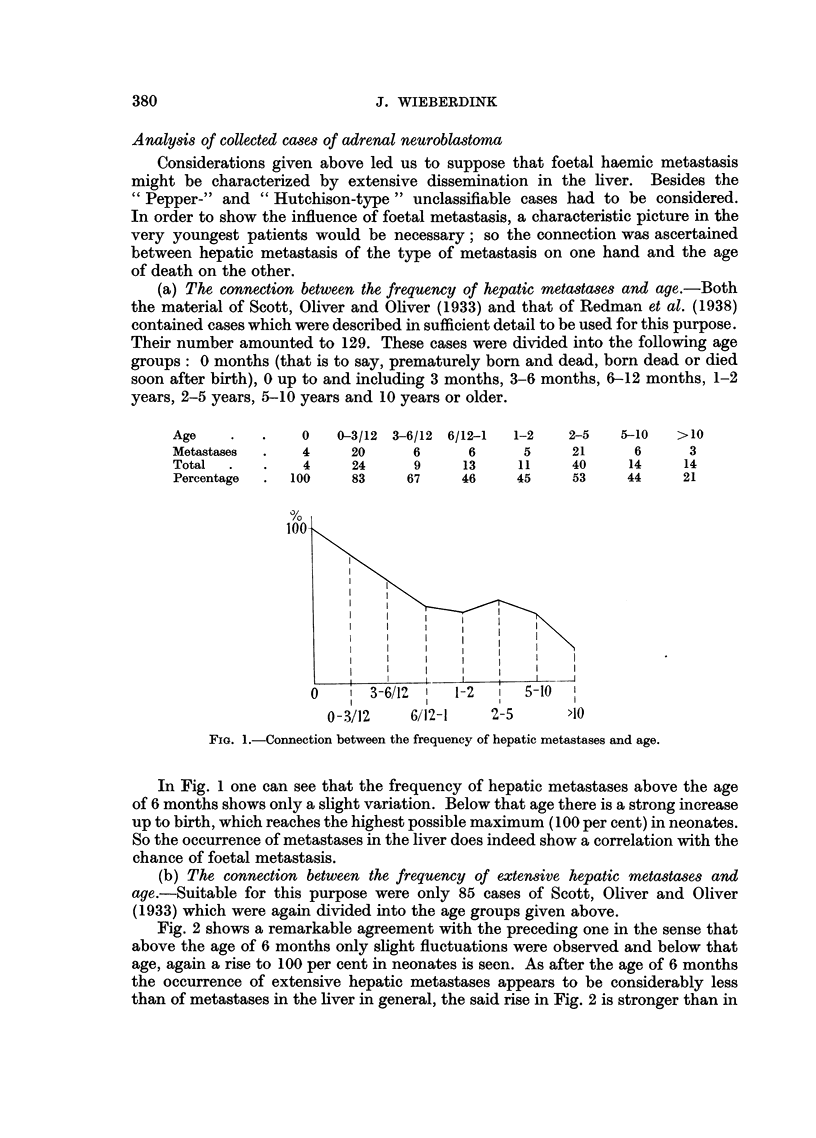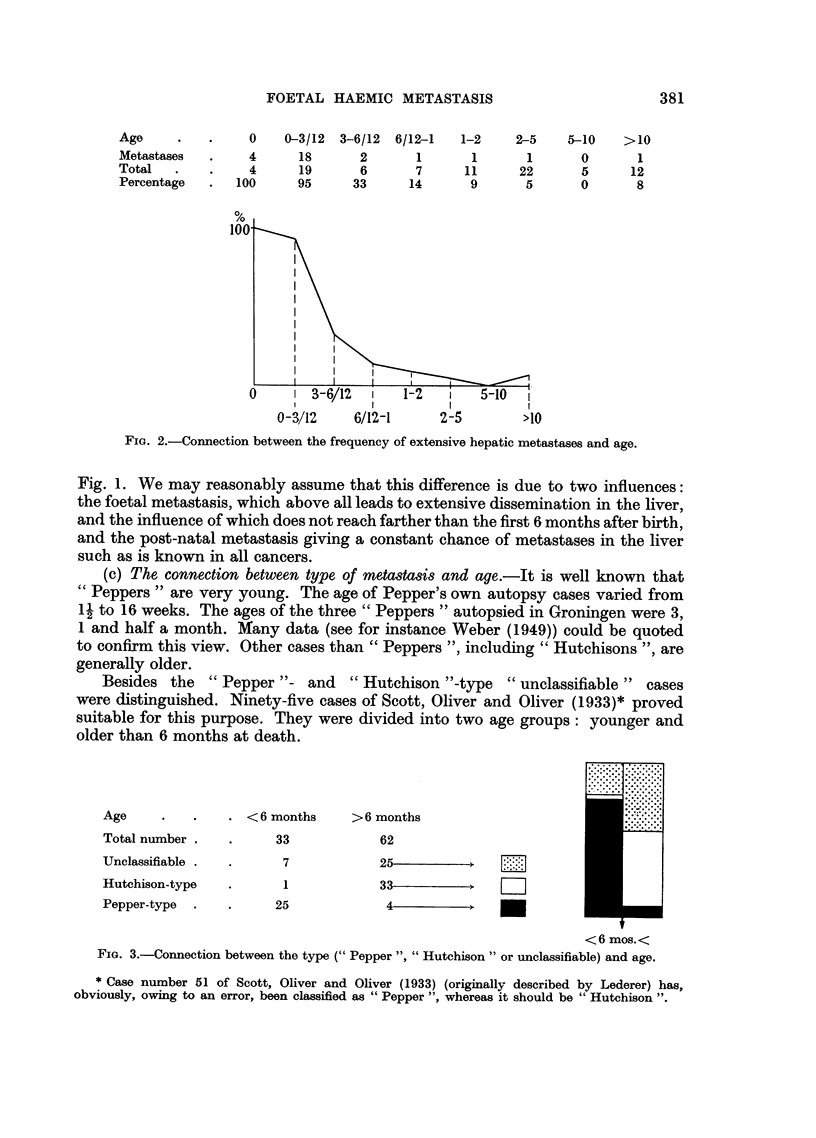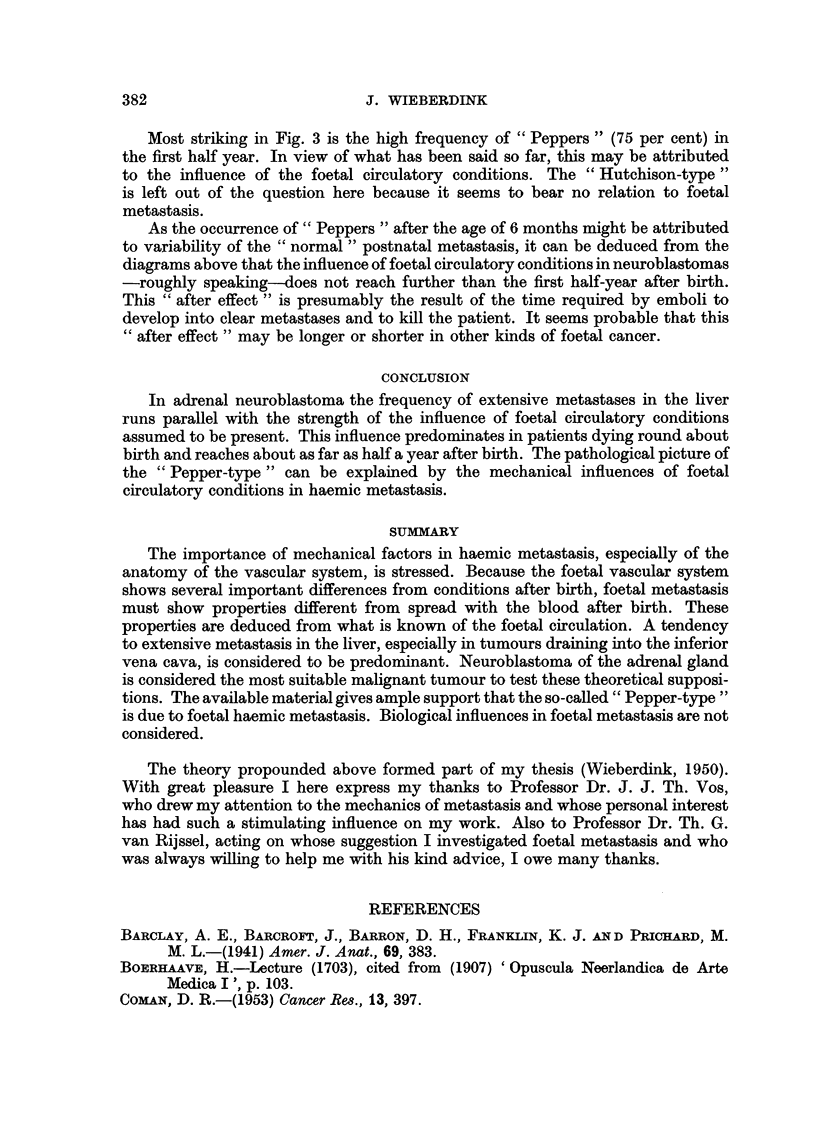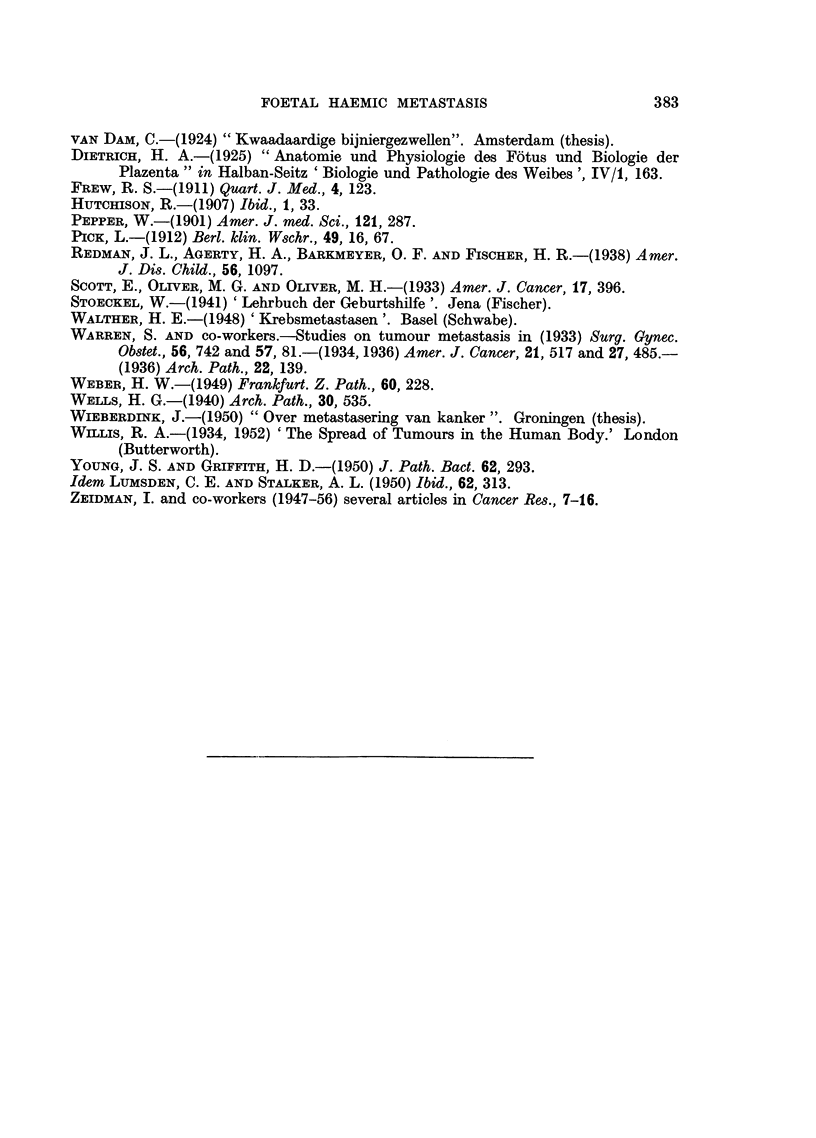# Foetal Haemic Metastasis. An Explanation of the “Pepper-Type” of Metastasis in Adrenal Neuroblastoma

**DOI:** 10.1038/bjc.1957.44

**Published:** 1957-09

**Authors:** J. Wieberdink


### FOETAL HAEMIC METASTASIS. AN EXPLANATION OF THE "PEPPER-TYPE" OF METASTASIS IN ADRENAL NEUROBLASTOMA

J. WIEBERDINK

*From the Pathological Laboratory, University of Groningen, Netherlands*

Received for publication May 30, 1957

PROBLEMS pertaining to the science of medicine can be divided into two classes, mechanical and biological. Although biological ways of thought are necessary for a final understanding, the opinion of Boerhaave (1703) " that mechanics are exceedingly important for, and absolutely indispensable to, the science of medicine" is still applicable today.

In metastasis of cancer the importance of mechanics is shown by the routes of spread, which determine to a great extent the location of seedlings. Broadly speaking, metastases in lymph nodes are of lymphatic, metastases in organs of haemic origin. Mechanical influences in haemic metastasis were experimentally investigated by Coman (1953), Warren (1933–1936), Young and Griffith (1950), Young, Lumsden and Stalker (1950) and Zeidman and co-workers (1947–1956). Standard works on pathology of human metastasis were written by Willis (1934, 1952) and Walther (1948). Many other workers in this field must remain unmentioned here.

Generally, haemic metastasis begins in the venous part of the circulatory system. The spread of cancer cells ends in the capillaries of the organs, these acting to a great extent as filters for tumour emboli. So, the " linkage " of the organs in the circulatory system plays a decisive part in haemic metastasis. We considered that in this respect conditions before and after birth differ vastly. We supposed that this might result in peculiarities of metastasis in foetal tumours. This hypothesis has been the basis of the investigation reported below.

*Anatomical peculiarities of the foetal vascular system.*

There are three important points of difference between the vascular anatomy before and after birth.

(a) *The short circuits of the pulmonary circulation via foramen ovale and ductus arteriosus.*—These short circuits can convey tumour emboli directly from the venous into the arterial part of the systemic circulation, thus avoiding arrest in the lungs. The ductus Botalli reaches the aorta distal to the branches to myocardium, head, neck and arms. Therefore, these parts of the body receive " unfiltered " blood only via the foramen ovale, whereas trunk, intestines, legs and placenta receive unfiltered blood via both short circuits. In this connection it is of importance to know what percentage of the blood in the inferior vena cava (coming from adrenal glands, liver, placenta and other organs) passes outside the " lung-filter " into the descending aorta and has therefore a chance of reaching the umbilical arteries. Accurate data about this quantity in human foetuses are not available.

The nearest approach to the right answer is probably given by Barclay *et al.* (1941). Using their estimates, it appears that at least half the blood from the inferior vena cava passes unfiltered into the descending aorta.

(b) *The foetal placental circulation.*—According to Dietrich (1925) the pressure in the umbilical arteries at the end of pregnancy varies from 50 to 83 mm., and the pressure in the umbilical vein from 32 to 48 mm. of mercury, roughly a drop from 2 to 1. In all other organs the drop from arterial to venous blood pressure is roughly from 100 to 1. It is clear from these figures that mechanical resistance in the placenta is exceptionally slight. Owing to wide connections between its arteries and veins, the placenta must be considered to be at the most a very coarse filter for corpuscular constituents of the foetal blood.

(c) *The ductus venosus Arantii.*—Through this vessel a part of the blood from the placenta passes outside the liver directly to the inferior vena cava. According to Grosser (cited by Stoeckel, 1941) the part that goes through the liver relatively increases during foetal life, so that this finally amounts to more than half.

From these considerations it may be deduced that foetal malignant tumours of organs with venous drainage to the inferior vena cava have little chance of giving rise to metastases in the lungs. Via the short circuits of the pulmonary circulation tumour emboli may easily be conveyed to the descending aorta. Once here they have a good chance of passing through the placenta to the liver. Deposits in this organ will in their turn, have a strong tendency to metastasize into the liver, because the hepatic vein is one of the branches of the inferior vena cava. Compared with metastasis after birth, foetal dissemination may be expected to be characterized by extensive hepatic metastases, especially if the primary tumour is located in the drainage area of the inferior vena cava.

*The choice of adrenal neuroblastoma for the investigation of foetal metastasis*

From Wells' (1940) valuable article on congenital malignant neoplasms it was deduced that adrenal neuroblastoma best fulfilled our requirements : occurring with sufficient frequency both before and after birth, distinct primary location and tendency to haemic dissemination. Only those cases were accepted in which the picture at autopsy was not essentially influenced by therapeutic measures. For this reason we confined ourselves to the autopsy cases collected by Scott, Oliver and Oliver (1933) and of Redman *et al.* (1938), as most of the more recent publications deal with intensively treated cases.

It is well known that by the beginning of this century neuroblastoma of the adrenal gland had already attracted the attention of pathologists by its ways of metastasis. The most characteristic feature of the " *Pepper-type* " is the excessive enlargement of the liver (Pepper, 1901). In the " *Hutchison-type* ", on the other hand, metastases in the skeleton (skull) predominate (Hutchison, 1907). It is beyond the scope of this article to reproduce all attempts in the literature to explain these types. None of them (for instance those given by Frew, (1911), Pick (1912) and van Dam (1924) has proved satisfactory. Several authors refuse to attach any special importance to these types of metastasis, or give them a definition deviating completely from the original descriptions. This too cannot be discussed in detail here. Suffice it to say that study of literature as well as experiences in the pathological laboratory at Groningen (3 unmistakable " Peppers " and 1 " Hutchison ") have led us to the opinion that the " Pepper-type " and, in a lesser degree, the " Hutchison-type " are too numerous to be ascribed to mere coincidence.

*Analysis of collected cases of adrenal neuroblastoma*

Considerations given above led us to suppose that foetal haemic metastasis might be characterized by extensive dissemination in the liver. Besides the " Pepper-" and " Hutchison-type " unclassifiable cases had to be considered. In order to show the influence of foetal metastasis, a characteristic picture in the very youngest patients would be necessary ; so the connection was ascertained between hepatic metastasis of the type of metastasis on one hand and the age of death on the other.

(a) *The connection between the frequency of hepatic metastases and age.*—Both the material of Scott, Oliver and Oliver (1933) and that of Redman *et al.* (1938) contained cases which were described in sufficient detail to be used for this purpose. Their number amounted to 129. These cases were divided into the following age groups : 0 months (that is to say, prematurely born and dead, born dead or died soon after birth), 0 up to and including 3 months, 3–6 months, 6–12 months, 1–2 years, 2–5 years, 5–10 years and 10 years or older.

| Age | 0 | 0–3/12 | 3–6/12 | 6/12–1 | 1–2 | 2–5 | 5–10 | >10 |
|---|---|---|---|---|---|---|---|---|
| Metastases | 4 | 20 | 6 | 6 | 5 | 21 | 6 | 3 |
| Total | 4 | 24 | 9 | 13 | 11 | 40 | 14 | 14 |
| Percentage | 100 | 83 | 67 | 46 | 45 | 53 | 44 | 21 |

FIG. 1.—Connection between the frequency of hepatic metastases and age.

In Fig. 1 one can see that the frequency of hepatic metastases above the age of 6 months shows only a slight variation. Below that age there is a strong increase up to birth, which reaches the highest possible maximum (100 per cent) in neonates. So the occurrence of metastases in the liver does indeed show a correlation with the chance of foetal metastasis.

(b) *The connection between the frequency of extensive hepatic metastases and age.*—Suitable for this purpose were only 85 cases of Scott, Oliver and Oliver (1933) which were again divided into the age groups given above.

Fig. 2 shows a remarkable agreement with the preceding one in the sense that above the age of 6 months only slight fluctuations were observed and below that age, again a rise to 100 per cent in neonates is seen. As after the age of 6 months the occurrence of extensive hepatic metastases appears to be considerably less than of metastases in the liver in general, the said rise in Fig. 2 is stronger than in

| Age | 0 | 0–3/12 | 3–6/12 | 6/12–1 | 1–2 | 2–5 | 5–10 | >10 |
|---|---|---|---|---|---|---|---|---|
| Metastases | 4 | 18 | 2 | 1 | 1 | 1 | 0 | 1 |
| Total | 4 | 19 | 6 | 7 | 11 | 22 | 5 | 12 |
| Percentage | 100 | 95 | 33 | 14 | 9 | 5 | 0 | 8 |

Fig. 2.—Connection between the frequency of extensive hepatic metastases and age.

Fig. 1. We may reasonably assume that this difference is due to two influences: the foetal metastasis, which above all leads to extensive dissemination in the liver, and the influence of which does not reach farther than the first 6 months after birth, and the post-natal metastasis giving a constant chance of metastases in the liver such as is known in all cancers.

(c) *The connection between type of metastasis and age.*—It is well known that " Peppers " are very young. The age of Pepper's own autopsy cases varied from $1\frac{1}{2}$ to 16 weeks. The ages of the three " Peppers " autopsied in Groningen were 3, 1 and half a month. Many data (see for instance Weber (1949)) could be quoted to confirm this view. Other cases than " Peppers ", including " Hutchisons ", are generally older.

Besides the " Pepper "- and " Hutchison "-type " unclassifiable " cases were distinguished. Ninety-five cases of Scott, Oliver and Oliver (1933)* proved suitable for this purpose. They were divided into two age groups : younger and older than 6 months at death.

| Age | <6 months | >6 months |
|---|---|---|
| Total number | 33 | 62 |
| Unclassifiable | 7 | 25 |
| Hutchison-type | 1 | 33 |
| Pepper-type | 25 | 4 |

Fig. 3.—Connection between the type (" Pepper ", " Hutchison " or unclassifiable) and age.

* Case number 51 of Scott, Oliver and Oliver (1933) (originally described by Lederer) has, obviously, owing to an error, been classified as " Pepper ", whereas it should be " Hutchison ".

Most striking in Fig. 3 is the high frequency of " Peppers " (75 per cent) in the first half year. In view of what has been said so far, this may be attributed to the influence of the foetal circulatory conditions. The " Hutchison-type " is left out of the question here because it seems to bear no relation to foetal metastasis.

As the occurrence of " Peppers " after the age of 6 months might be attributed to variability of the " normal " postnatal metastasis, it can be deduced from the diagrams above that the influence of foetal circulatory conditions in neuroblastomas —roughly speaking—does not reach further than the first half-year after birth. This " after effect " is presumably the result of the time required by emboli to develop into clear metastases and to kill the patient. It seems probable that this " after effect " may be longer or shorter in other kinds of foetal cancer.

### CONCLUSION

In adrenal neuroblastoma the frequency of extensive metastases in the liver runs parallel with the strength of the influence of foetal circulatory conditions assumed to be present. This influence predominates in patients dying round about birth and reaches about as far as half a year after birth. The pathological picture of the " Pepper-type " can be explained by the mechanical influences of foetal circulatory conditions in haemic metastasis.

### SUMMARY

The importance of mechanical factors in haemic metastasis, especially of the anatomy of the vascular system, is stressed. Because the foetal vascular system shows several important differences from conditions after birth, foetal metastasis must show properties different from spread with the blood after birth. These properties are deduced from what is known of the foetal circulation. A tendency to extensive metastasis in the liver, especially in tumours draining into the inferior vena cava, is considered to be predominant. Neuroblastoma of the adrenal gland is considered the most suitable malignant tumour to test these theoretical suppositions. The available material gives ample support that the so-called " Pepper-type " is due to foetal haemic metastasis. Biological influences in foetal metastasis are not considered.

The theory propounded above formed part of my thesis (Wieberdink, 1950). With great pleasure I here express my thanks to Professor Dr. J. J. Th. Vos, who drew my attention to the mechanics of metastasis and whose personal interest has had such a stimulating influence on my work. Also to Professor Dr. Th. G. van Rijssel, acting on whose suggestion I investigated foetal metastasis and who was always willing to help me with his kind advice, I owe many thanks.

### REFERENCES

Barclay, A. E., Barcroft, J., Barron, D. H., Franklin, K. J. and Prichard, M. M. L.—(1941) *Amer. J. Anat.*, **69**, 383.

Boerhaave, H.—Lecture (1703), cited from (1907) ' Opuscula Neerlandica de Arte Medica I ', p. 103.

Coman, D. R.—(1953) *Cancer Res.*, **13**, 397.

van Dam, C.—(1924) " Kwaadaardige bijniergezwellen". Amsterdam (thesis).
Dietrich, H. A.—(1925) " Anatomie und Physiologie des Fötus und Biologie der Plazenta " *in* Halban-Seitz ' Biologie und Pathologie des Weibes ', IV/1, 163.
Frew, R. S.—(1911) *Quart. J. Med.*, **4**, 123.
Hutchison, R.—(1907) *Ibid.*, **1**, 33.
Pepper, W.—(1901) *Amer. J. med. Sci.*, **121**, 287.
Pick, L.—(1912) *Berl. klin. Wschr.*, **49**, 16, 67.
Redman, J. L., Agerty, H. A., Barkmeyer, O. F. and Fischer, H. R.—(1938) *Amer. J. Dis. Child.*, **56**, 1097.
Scott, E., Oliver, M. G. and Oliver, M. H.—(1933) *Amer. J. Cancer*, **17**, 396.
Stoeckel, W.—(1941) ' Lehrbuch der Geburtshilfe '. Jena (Fischer).
Walther, H. E.—(1948) ' Krebsmetastasen '. Basel (Schwabe).
Warren, S. and co-workers.—Studies on tumour metastasis in (1933) *Surg. Gynec. Obstet.*, **56**, 742 and **57**, 81.—(1934, 1936) *Amer. J. Cancer*, **21**, 517 and **27**, 485.—(1936) *Arch. Path.*, **22**, 139.
Weber, H. W.—(1949) *Frankfurt. Z. Path.*, **60**, 228.
Wells, H. G.—(1940) *Arch. Path.*, **30**, 535.
Wieberdink, J.—(1950) " Over metastasering van kanker ". Groningen (thesis).
Willis, R. A.—(1934, 1952) ' The Spread of Tumours in the Human Body.' London (Butterworth).
Young, J. S. and Griffith, H. D.—(1950) *J. Path. Bact.* **62**, 293.
*Idem* Lumsden, C. E. and Stalker, A. L. (1950) *Ibid.*, **62**, 313.
Zeidman, I. and co-workers (1947–56) several articles in *Cancer Res.*, **7–16**.